# TNFα-Mediated Loss of β-Catenin/E-Cadherin Association and Subsequent Increase in Cell Migration Is Partially Restored by NKX3.1 Expression in Prostate Cells

**DOI:** 10.1371/journal.pone.0109868

**Published:** 2014-10-31

**Authors:** Bilge Debelec-Butuner, Cansu Alapinar, Nursah Ertunc, Ceren Gonen-Korkmaz, Kutsal Yörükoğlu, Kemal Sami Korkmaz

**Affiliations:** 1 Department of Bioengineering, Cancer Biology Laboratory, Faculty of Engineering, Ege University, Bornova, Izmir, Turkey; 2 Department of Pharmaceutical Biotechnology, Faculty of Pharmacy, Ege University, Bornova, Izmir, Turkey; 3 Department of Pharmacology, Ege University, Bornova, Izmir, Turkey; 4 Department of Pathology, Faculty of Medicine, Dokuz Eylul University, Inciralti, Izmir, Turkey; National Institutes of Health, United States of America

## Abstract

Inflammation-induced carcinogenesis is associated with increased proliferation and migration/invasion of various types of tumor cells. In this study, altered β-catenin signaling upon TNFα exposure, and relation to loss of function of the tumor suppressor NKX3.1 was examined in prostate cancer cells. We used an *in vitro* prostate inflammation model to demonstrate altered sub-cellular localization of β-catenin following increased phosphorylation of Akt^(S473)^ and GSK3β^(S9)^. Consistently, we observed that subsequent increase in β-catenin transactivation enhanced c-myc, cyclin D1 and MMP2 expressions. Consequently, it was also observed that the β-catenin-E-cadherin association at the plasma membrane was disrupted during acute cytokine exposure. Additionally, it was demonstrated that disrupting cell-cell interactions led to increased migration of LNCaP cells in real-time migration assay. Nevertheless, ectopic expression of NKX3.1, which is degraded upon proinflammatory cytokine exposure in inflammation, was found to induce the degradation of β-catenin by inhibiting Akt^(S473)^ phosphorylation, therefore, partially rescued the disrupted β-catenin-E-cadherin interaction as well as the cell migration in LNCaP cells upon cytokine exposure. As, the disrupted localization of β-catenin at the cell membrane as well as increased Akt^(S308)^ priming phosphorylation was observed in human prostate tissues with prostatic inflammatory atrophy (PIA), high-grade prostatic intraepithelial neoplasia (H-PIN) and carcinoma lesions correlated with loss of NKX3.1 expression. Thus, the data indicate that the β-catenin signaling; consequently sub-cellular localization is deregulated in inflammation, associates with prostatic atrophy and PIN pathology.

## Introduction

Due to its high occurrence in the western world, prostate cancer constitutes a major health problem [1]. Therefore, there is remarkable interest in studying the molecular mechanisms responsible for the initiation, progression and metastasis of prostate cancer (PCa). Regardless of the etiology, inflammation is reported as one of the major contributors to cancer development. Acute and/or chronic inflammation occur in tissues and alter signal transduction pathways, including Akt and Wnt/β-catenin, to contribute to neoplastic transformation [2]. However, during the inflammatory response, activated macrophages secrete many glycoproteins that may enhance Akt-mediated survival and consequently facilitate the transactivation of β-catenin [3], which is a well-known transcriptional regulator of Wnt signaling [4].

β-catenin is a dual-function protein that is an important component of the plasma membrane and plays a central role in cell-cell adhesion. Accordingly, solid tumors, including those of the prostate, frequently display considerable β-catenin accumulation [2] and this β-catenin accumulation plays a significant role in prostate carcinogenesis by contributing to uncontrolled cell proliferation and differentiation [5]. In the absence of a Wnt signal, β-catenin is targeted for proteosomal degradation via ubiquitination following phosphorylation by the cytoplasmic Axin/GSK3β/APC (destruction) complex. The S33, S37, T41 and S45 phosphorylation sites in β-catenin regulate its abundance in the cytoplasm by controlling the stabilization or degradation of the protein [6], [7]. However, increased Wnt signaling and activating phosphorylation of Akt^(S473)^ results in inhibitory phosphorylation of GSK3β^(S9)^. The inhibition of GSK3β suppresses the phosphorylation of β-catenin at S33, ultimately resulting in its cytoplasmic accumulation. Eventually, a part of free β-catenin translocates to nucleus [5], [8] and activates the expression of its targets such as c-myc and cyclin D1, resulting in deregulated cell cycle progression.

During inflammation, mutations in components of the Wnt/β-catenin signaling pathway or induced secretion of Wnt glycoproteins from activated macrophages may thus result in stabilization of β-catenin. Regardless of Wnt signaling, inflammation-mediated increase in the transcriptional activity of β-catenin may also be enhanced via phosphorylation of β-catenin^(S552)^ by Akt kinase [5]. Thus, Akt activity leads to β-catenin stabilization by suppressing the GSK3β kinase, thereby preventing the proteosomal degradation of β-catenin, consequently results increased expression of β-catenin target genes [9].

During carcinogenesis, increased transcriptional activity of β-catenin correlates with the loss of E-cadherin-mediated cell adhesion [10], [11], which is an essential for calcium-dependent intercellular adhesion in adherent junctions [12]. Nevertheless, deregulation of the phosphorylation of β-catenin^(Y654)^ and ^(Y142)^ influence interactions with E-cadherin and α-catenin, respectively. Further, the loss of the interaction between β-catenin and E-cadherin enhances the transcriptional activity of β-catenin and promotes the epithelial-to-mesenchymal transition [11], ultimately increases the cell migration [8]. Therefore, the development of high migration rates during earlier stages of prostate carcinogenesis [13], [14] and growth factor activation under the inflammatory microenvironment needs to be examined.

NKX3.1 is an androgen-regulated tumor suppressor in prostate cells [15], [16] and is reported to have an important role in DNA damage regulation [17], [18]. The loss of NKX3.1 expression was reported as a common event in high-grade prostate tumors [17]. It has previously been reported that the pro-inflammatory cytokines TNFα and IL-1β induce proteosomal degradation of NKX3.1 [19], [20]. Furthermore, when Nkx3.1 expression was examined in infected prostate lobes in mice, it was found that diminished AR expression correlated with the loss of Nkx3.1 expression [21]. Moreover, NKX3.1 and androgen receptor (AR) ubiquitination and their subsequent proteosomal degradation were reported after inflammation, and revealed that the loss of NKX3.1 expression was not directly related to the loss of the AR transactivating function [19], [20]. Consistent with previous findings, the decreased NKX3.1 expression level was related to cytokine exposure; the major cytokine associated with the loss of NKX3.1 in our studies was TNFα. Accordingly, inflammatory cytokines were shown to contribute to a deregulated apoptotic response in prostate cells with a loss of the growth-regulatory function of AR, leading to increased proliferation during inflammation [20].

In this study, using the prostate cancer cell line (LNCaP), the mechanism underlying inflammation-induced tumorigenesis, β-catenin localization change and E-cadherin association, and the role of NKX3.1 in these processes were investigated. First, a tightly controlled cytokine exposure that partially mimics prostatic inflammatory atrophy [20] was used to study the acute (500 pg/ml TNFα, up to 24 h) and chronic (62 or 125 pg/ml TNFα, for 4 weeks) inflammatory microenvironment in LNCaP cells. Second, human prostate tissues from 14 patients with prostatic inflammatory disease (and cancer) were examined. Then, we found that some of the atrophic glands inherited PIN lesions in close proximity to adenocarcinomas, which exhibited a substantial loss of membrane-bound β-catenin. These samples also exhibited a clear loss of NKX3.1 expression in these atrophic glands. Thus, we suggest that an exposure to prostatic inflammation-like microenvironment facilitates the progression of prostate cancer via increased β-catenin stabilization presumably from the PIA in glands, subsequent to the loss of function of cell cycle regulators such as NKX3.1 that is required for intact tissue organization and cell cycle regulation.

## Materials and Methods

### Macrophage Differentiation and Conditioned Media (CM) Collection

The U937 monocyte cell line was cultured in RPMI 1640 medium including 10% FBS (fetal bovine serum) at 37°C in the presence of 5% CO_2_. To achieve macrophage differentiation and cytokine production, cells (8×10^5^) were seeded into 75 cm^2^ culture flasks for 2 h prior to the treatments. Next, PMA was added at final concentrations of 2, 4, 8 and 16 nM for 16 h, and the adherent clusters were followed. The cells were washed twice, and 20 ml of fresh medium was added. After allowing the cells to rest for 3 h, lipopolysaccharide (LPS) was added at a final concentration of 10 ng/ml, and the cells were incubated for 24 h. Finally, the supernatant (conditioned medium - CM) was collected and filtered (using a 0.22 µm filter) for further use.

### Measurement of Cytokines in the CM

Before feeding the LNCaP cells with the collected CM, TNFα (Invitrogen, Carlsbad, US), interleukin-6 (IL6) and interleukin-1beta (IL1β) (Boster Biological Technology Co., US) levels were examined using an ELISA according to the manufacturer's instructions. Because exposure is a major component in our inflammation model, the time (0, 2, 4, 6, 12 and 24 h) and the dose (62.5, 125, 250 or 500 pg/ml TNFα-containing CM) for the courses of CM treatments were optimized separately. Ultimately, TNFα was chosen as a measure of CM exposure.

### Cell Culture and Treatments

LNCaP cells were obtained from the American Type Culture Collection (ATCC, Manassas, VA) and were propagated as recommended using RPMI 1640 medium supplemented with 10% FBS, L-glutamine (2 mM), penicillin (100 U/ml) and streptomycin (100 µg/ml) at 37°C in the presence of 5% CO_2_. The acute inflammation treatments were performed with CM (500 pg/ml TNFα) at appropriate periods of 6 or 24 h. In chronic inflammation model, cells were treated with 62 or 125 pg/ml TNFα-containing CM for 4 weeks. TNFα concentrations were adjusted by diluting the CM using RPMI 1640 medium as described previously [20].

### Transfections

The *NKX3.1* open reading frame was amplified (using the primers F: GGATCCATGCTCAGGGTTCCGGAGCCG and R: GAATTCGGTTGTCACCTGAGCTGGCATTA) and cloned into the pcDNA4/HisMax-TOPO vector (Invitrogen, USA) according to the manufacturer's instructions. The transfections were performed using the Fugene HD transfection reagent (Roche, Germany) for 24 h. Briefly; 4×10^5^ cells were seeded into 6 cm plates. Next day, medium was changed to serum-free medium (SFM), and the transfection mix was prepared by adding 3 µl of Fugene HD into 100 µl of SFM. Then, the mix was incubated for 5 min at RT, 1 µg of DNA was added, and 15 min later, the transfection mix was added onto cells dropwise. After 6 h of incubation, pre-warmed RPMI medium including 2× FBS was added, and the cells were incubated for an additional 18 to 42 h.

### Antibodies

The following antibodies were purchased from manufacturers: β-catenin, p-β-catenin^(S33)^, p-Akt^(S473)^, p-Akt^(S308)^, p-GSK3β^(S9)^, c-myc, cyclin D1, E-cadherin, NKX3.1 and poly-ubiquitin (Santa Cruz Inc., Germany), p-β-catenin^(S552)^, Matrix metalloproteinase 2 (MMP2) (Cell Signaling, USA) and Glucose 6 phosphate dehydrogenase (GAPDH) (Ambion, UK). The NKX3.1 custom antibody was a gift from Dr. F. Saatcioglu (University of Oslo). The HRP-conjugated anti-mouse and anti-rabbit (Amersham, UK) and Alexa Fluor 488- and 594-conjugated (Invitrogen, CA) secondary antibodies were purchased and used as recommended.

### Cell Lysis, Protein Extraction and Blotting

For protein extraction, cells were grown in 6 cm culture dishes (Sarstedt, Germany) and washed once with PBS prior to cell lysis. Cells were resuspended in 250 µl of modified RIPA buffer (10 mM Tris.Cl (pH: 8.0), 1% Triton X-100, 0.1% SDS, 0.1% Na deoxycholate, 1 mM EDTA, 1 mM EGTA, 140 mM NaCl) containing protease and phosphatase inhibitors. Cells were collected from culture plates using a cell scraper and transferred to eppendorf tubes. Lysates were sonicated for 20 seconds (25% power, 0.5 cycles), centrifuged at 12000 g for 10 minutes, and cleared supernatants were collected into new tubes. Protein concentrations were determined using BCA assay (Sigma, UK). SDS-PAGE and western blots were performed under standard conditions using 50 µg of protein lysate per lane; proteins were separated on a 10–12% gel and transferred to PVDF membrane (Amersham, UK) using a wet transfer blotter. The PVDF membrane was blocked with 5% dry milk in TBS-T (Tris-Borate-Saline solution containing 0.1% Tween 20). Primary and secondary antibody incubations were performed using TBS-T containing 0.5% dry milk or 5% BSA at RT for 1 h or at 4°C for o/n. Membranes were developed using ECL plus reagent (Amersham, UK) for 5 min and were photographed using Kodak X-Ray films in a dark room.

### Sub-Cellular Fractionation

LNCaP cells (2×10^7^) were washed with PBS and pelleted for 5 min at 300 g. The cell pellet was resuspended in 500 µl buffer A (250 mM sucrose, 50 mM Tris-HCl, 5 mM MgCl_2_) and cell lysis was performed by sonication on ice (3 times 10 sec. pulse with 40% power and 30 sec. interval). The suspension was centrifuged at 800 g for 15 min and the pellet A was saved to isolate nuclei. The supernatant A was centrifuged again at 1000 g for 15 min. The supernatant B was saved to isolate the cytosolic proteins. The pellet A, saved for isolation of nuclei, was dissolved in 500 µl buffer A, and centrifuged at 1000 g for 15 min. The supernatant C was added to the supernatant B for isolating cytosolic proteins and stored on ice until then. The pellet C was resuspended in 500 µl buffer B1 (1 M sucrose, 50 mM Tris-HCl, 5 mM MgCl_2_) and layered onto a 1.5 ml cushion of buffer B2 (2 M sucrose, 50 mM Tris-HCl, 5 mM MgCl_2_). Then, centrifugation at 2100 g for 1 h was done. The pellet D was taken up in 250 µl buffer D (20 mM HEPES, pH: 7.9, 1.5 mM MgCl_2_, 0.5 M NaCl, 0.2 mM EDTA, 20% glycerol, 1% Triton X-100) and incubated for 1 h by rotating at 30 rpm. The suspension was sonicated on ice again (3 times 10 sec. pulses with 40% power and 30 sec. interval) and centrifuged at 9000 g for 30 min. **nuclear** protein lysate was collected as supernatant. Furthermore, the pooled supernatants B and C were centrifuged for 150 min at 16000 g to isolate **cytosolic** protein lysate from supernatant. The pellet was dissolved in 250 µl buffer C (20 mM Tris-HCl, 0.4 M NaCl, 15% glycerol, 1.5% Triton X-100), incubated 1 h by rotating at 30 rpm and centrifuged at 9000 g for 30 min, to isolate **membrane** components from supernatant. All incubations and centrifugations were carried out at 4°C, buffers were supplemented with 1× protease inhibitor cocktail (Roche GmbH, Germany) and 1 mM DTT directly before use. Fifty µg of each fraction was subjected to SDS-PAGE.

### Immunofluorescence Labeling and Microscopy

Cells were grown on coverslips and CM treatment was performed. At the time of analysis, cells on coverslips were rinsed with PBS, fixed with methanol at −20°C for 30 min, permeabilized with 0.2% triton X-100 in PBS for 5 min on a shaker and blocked for 5 min using 1% BSA in PBS. Primary antibodies (in 1% BSA/PBS) were added and incubated in a humidified chamber for 1 h, and cells were washed twice with PBS. Secondary antibody incubations were performed at RT for 20 min using Alexafluor 488 (anti-rabbit) and/or Alexafluor 594 (anti-mouse) antibodies. Finally, cells were washed twice with PBS and mounted on coverslips with 30% glycerol in PBS including 0.5 µg/ml DAPI, and analyzed immediately using Leica DM4000B LED fluorescent microscope (Leica, Germany). Images were captured using Leica imaging software.

### Real-Time Proliferation and Migration Assay

The Xcelligence migration and proliferation assay system was used for real time measurements. Briefly, the LNCaP cells (8×10^3^) were untransfected or transfected with the HM control vector and HM-NKX3.1 (24 h), seeded into 96-well plates (E-plates, Roche GmbH, Germany) and grown for 24 h. CM treatments were performed, and the growth rate and morphological changes were followed. The data (impedance values) were collected every 10 min for an additional 48 h. For the migration assay, 8×10^3^ cells were seeded into 16-well plates (CIM-plates, Roche GmbH, Germany). FBS was used as a chemo-attractant, and the migration rate was followed. The data were collected every 10 min for an additional 136 h.

### WST Growth Assay

LNCaP (2×10^5^) cells were seeded and grown in 6-well plates for different time points (0, 3, 6, 24 h). At the end of treatments, WST formazan dye assay was performed for cells grown in cultures.

### Immunoprecipitation

The protein lysate (500 µg) was subjected to immunoprecipitation using 2 µg of the β-catenin antibody at 4°C overnight. After the incubation, the antigen-antibody complexes were collected using 20 µl of Protein A/G plus agarose beads (Santa Cruz Inc., USA) for 1 h at 4°C. After centrifugation at 4000×g for 5 min, the pellets were washed with 500 µl of modified RIPA buffer 5 times. The collected beads were resuspended in 20 µl of RIPA buffer, and half of each sample was used for the SDS-PAGE analysis.

### Immunohistochemistry

The tissue blocks containing the most representative areas from 14 radical prostatectomy specimens (containing normal, high grade prostatic intraepithelial neoplasia [H-PIN] and adenocarcinoma) were chosen based on H&E staining, and 5 µm sections were cut and mounted on poly-L-lysine-coated slides for immunohistochemical staining. A standard streptavidin-biotin immunoperoxidase method was used to stain the tissue sections with ß-catenin and NKX3.1 antibodies (Santa Cruz Biotechnology, dilution 1/200). Briefly, the tissue sections were treated in xylene, rehydrated in an alcohol series and immersed in distilled water. Endogenous peroxidase activity was blocked using a 0.3% solution of hydrogen peroxide in phosphate-buffered saline at room temperature (RT) for 10 min, and the sections were then rinsed with washing buffer (50 mM Tris-Cl, pH 7.5). The primary antibodies were applied for 1 h at RT, the sections were washed, the streptavidin-labeled peroxidase-conjugated antibody (Invitrogen, Histostain Plus, 85–9043) was added at RT for 10 min, and the sections were washed again. The peroxidase activity was visualized with 0.03% 3,3-diaminobenzidine tetrahydrochloride (DAB) (Sigma Chemical Co., St. Louis, Missouri, USA) for 5 min. The sections were then washed in deionized water, counterstained with Mayer's hematoxylin and mounted.

### Ethics Approval of Human Samples

Dokuz Eylul University, Medical Faculty's institutional ethics committee approved this study (one of the members of the board Prof. Dr. Kutsal Yorukoglu is the coauthor to this publication). The same committee waived the need for consent, as the same samples were previously used in another study [22].

### Statistics

The images were analyzed using image J software. Briefly, expression intensity measurements were performed individually for each nuclear and cytoplasmic area in pre-defined boundaries, which were selected in equal area^square^. Mean intensity _(nucleus/cytop.)_  =  mean intensity _(nucleus/cytop.)_ – mean background. Ratio  =  Mean intensity _(nucleus)_/Mean intensity _(cytop.)_. The data are presented as the mean ± SEM (standard error mean values). The differences in the mean values between the groups were analyzed by a two-tailed Student's *t*-test, and *P*<0.05 was considered statistically significant.

## Results

### CM Induces LNCaP Cell Growth and Alters Cellular Morphology

**Figure 1:**
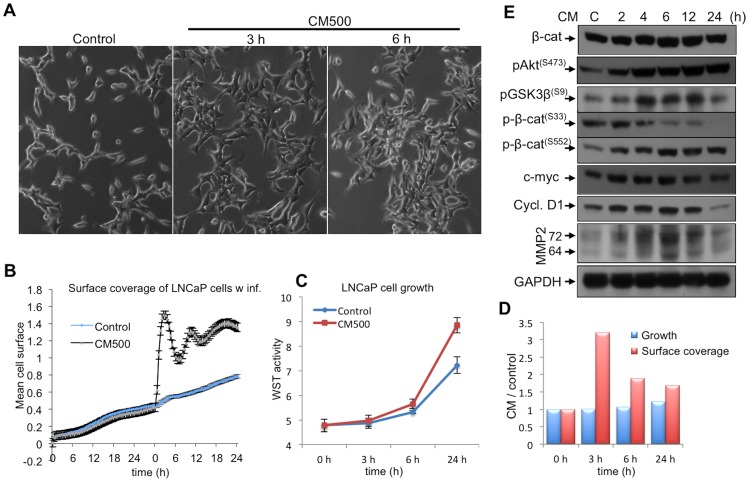
High dose (500 pg/ml) of TNFα disrupts LNCaP cell morphology. **A.** This was examined with regular phase/contrast microscopy (Scale bar represents 50 µm, and the magnification is 10x). **B.** When LNCaP cells were examined using a real-time cell proliferation assay, 3 to 6 h after the CM treatment (500 pg/ml TNFα), significant (p<0.001) augmentation of the cell surface area was observed. Real-time cell proliferation assay was performed twice in each 6 identical replicates and the western blots were performed at least twice as independent replicates. **C.** The growth is increased in CM treatments, **D.** whereas immediate (3 h) and remarkable cell morphology alterations occur in treatments comparison to controls. **E.** In CM treatment, β-catenin expression is upregulated besides increased Akt^(S473)^ phosphorylation. Also, inhibitory phosphorylation of GSK3β^(S9)^ and the stability-enhancing phosphorylation of β-catenin^(S552)^ increased concurrently to the p-β-catenin^(S33)^ decrease. As a result, the stabilized β-catenin-mediated transactivation increased the expression of c-myc, cyclin D1 and MMP2 that are shown.

To understand the role of the inflammatory microenvironment in prostate cells, optimized conditioned media (CM) including TNFα was added onto the LNCaP cells [20], and then the cellular alterations, cell surface coverage as well as growth were examined. At certain doses (250 and 500 pg/ml TNFα adjusted) and specific time points (3–6 h) of the CM treatments, an immediate and considerable response was observed in prostate cells (Figure 1A). To further characterize this response, cell growth was examined using a real-time proliferation assay, and the impedance readings from the growing cultures were converted into growth rate using the Xcelligence system. First, LNCaP cells were propagated for 24 h in a resting state, and then fed with CM. The impedance values were recorded real-time over 10 min intervals from each well. Thus, the treatments significantly changed the impedance corresponding to cell growth within 3 h (p<0.001) compared to untreated controls (Figure 1B). Nevertheless, as the observed rate of change (2.4-fold in 3 h) could not be due to the cellular growth and immediately occurred after CM treatment, we assumed that this could be a change in surface coverage of the cells and performed WST based assay measuring mitochondrial ATPase production (Figure 1C). The data suggest that an immediate change in cell morphology occurs upon CM exposure concurrent the increased cell growth (Figure 1D).

### An Increase in p-β-Catenin^(S552)^ by Enhanced Akt Activity Stabilizes β-Catenin after CM Exposure

To investigate the consequences of these changes in morphology of LNCaP cells, the components of β-catenin signaling pathway was studied after CM treatment. Specifically, CM-treated cells for growth, altered cell morphology and increased β-catenin stability at time points up to 24 h treatments were examined. Additionally, the molecular changes leading to β-catenin accumulation and phospho-specific changes that control the stability and transcriptional activity of β-catenin were also examined. As a result, we observed a significant increase in pAkt^(S473)^ upstream of β-catenin. As this phosphorylation event subsequently inhibited the GSK3β kinase by increasing the serine 9 phosphorylation, eventually resulted in a decrease in β-catenin^(S33)^ phosphorylation (Figure 1E). Since, it is well recognized that this modification is critical for regulating β-catenin turnover [8], we found that CM-mediated stabilization of β-catenin enhanced expression of the β-catenin transcriptional targets, c-myc, cyclin D1 and MMP2, evidenced in the western blots (Figure 1E). Therefore, we hypothesized that the degradation of β-catenin might be suppressed via CM treatments, consistent of an increase in β-catenin^(S552)^ phosphorylation, which is a direct target of Akt kinase.

### Membrane Localized β-Catenin Is Internalized with CM Treatment in LNCaP Cells

**Figure 2:**
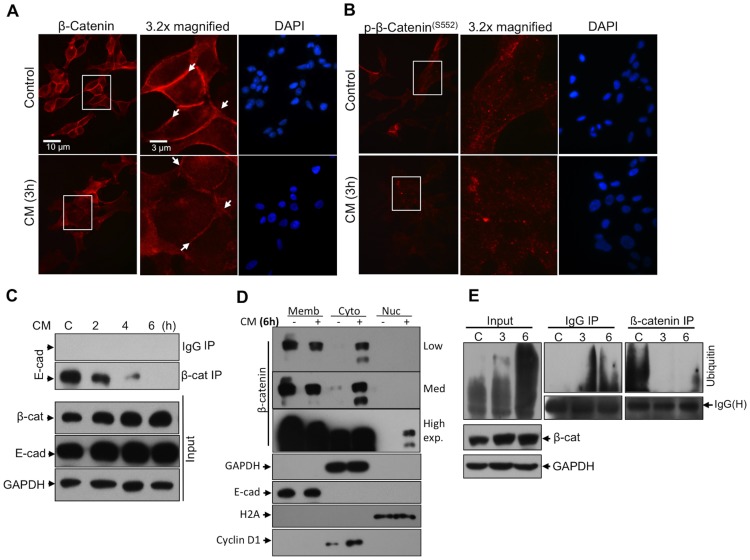
Inflammation influences the membrane-localized β-catenin and E-cadherin interaction. **A.** β-catenin and **B.** p-β-catenin^(S552)^ localizations at plasma membrane (arrows) are decreased with CM treatment (500 pg/ml TNFα for 3 h) (magnification 60X), and the β-catenin and p-β-catenin^(S552)^ localize into cell cytoplasm. **C.** The loss of membrane-localized β-catenin and E-cadherin interaction at the cell membrane was evidenced when immunoprecipitation (IP) time course was performed. **D.** A substantial increase in cytoplasmic and nuclear translocated β-catenin after CM treatments were also confirmed in the sub-cellular fractionated cell lysates. E-cadherin, H2A and GAPDH levels were also confirmed not changing after 6 h of treatment as controls for fractions. Memb: Membrane fraction, Nuc: Nuclear fraction, Cyto: Cytoplasmic fraction. **E.** Furthermore, total ubiquitination as well as β-catenin expression levels are increased in CM treated LNCaP cells whereas β-catenin ubiquitination is decreased. The antibodies for IPs were anti-mouse IgG or NKX3.1 and/or anti-β-catenin. The IPs and the blots were performed at least three times.

To determine the influence of inflammation in terms of β-catenin stabilization, subcellular localization of β-catenin, degree of phosphorylation at S552 residue and the interaction between β-catenin and E-cadherin were investigated using immunofluorescence microscopy and immunoprecipitation studies. First of all, we observed that the localization of β-catenin at membrane localizations was disrupted in LNCaP cells when they were treated with 500 pg/ml TNFα containing CM for 3 to 6 h (Figure 2A). Additionally, p-β-catenin^(S552)^ was increased at cytoplasm at 3 h of CM treatment (Figure 2B). Although the total E-cadherin and β-catenin expression levels remained similar in treatments (inputs in Figure 2C), we found an immediate and substantial loss in β-catenin-E-cadherin interaction after 3 h CM treatment (Figure 2C). To validate the increased transactivation of β-catenin by its altered subcellular localization, nuclear, cytoplasmic and membrane proteins were fractionated and β-catenin, E-cadherin, cyclin D1 and control proteins GAPDH and Histone 2 A (H2A) expressions were examined before and after CM treatments. Subcellular fractionation coupled western blotting revealed that β-catenin level increased substantially in cytoplasmic fraction after 6 h of CM treatment relative to GAPDH expression (Figure 2D). Consequently, β-catenin transactivation increased, which was confirmed via cyclin D1 expression (Figure 2D), though GAPDH, H2A and E-cadherin levels remained same. Finally, β-catenin ubiquitination was analyzed using IPs and displayed that β-catenin expression inversely correlated with ubiqitination of β-catenin in CM treated cells. Taken together, the data suggest that the β-catenin stabilization occurs due to inhibition of β-catenin degradation (Figure 2E).

### NKX3.1 Suppresses the Proliferative and Invasive Effects of Upregulated β-Catenin Signaling

**Figure 3:**
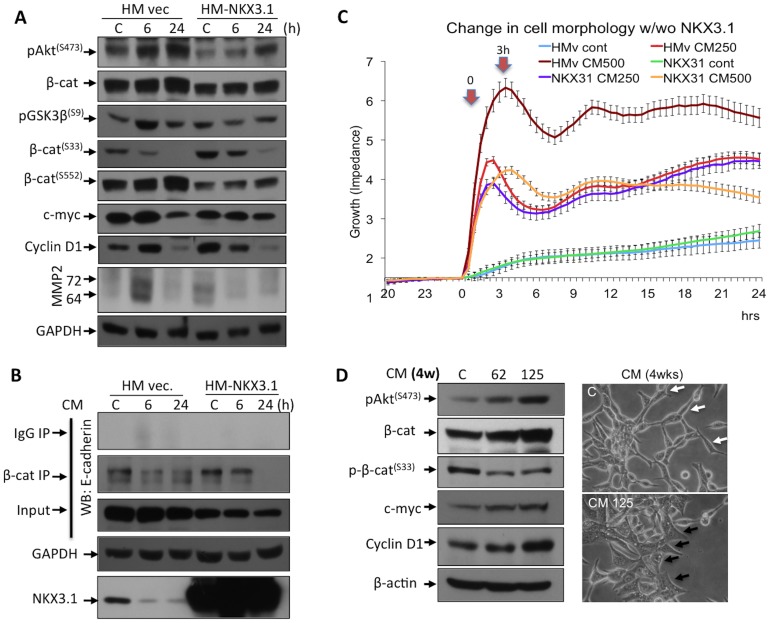
NKX3.1 suppressed the morphological changes and growth rate of the LNCaP cells, when the cells were treated with CM. **A.** Significant decreases in p-GSK3β^(S9)^ and p-β-catenin^(S552)^ and an increase in p-β-catenin^(S33)^ phosphorylation were observed after NKX3.1 overexpression. Consistently, c-myc, cyclin D1, and MMP2 expression levels were marginally decreased. **B.** Although, E-cadherin level is marginally decreased, NKX3.1 expression restored the β-catenin-E-cadherin interaction, which are disrupted by CM (500 pg/ml TNFα for 6 or 24 h) treatment in LNCaP cells. HM-vec and HM-NKX3.1 represent the control and the HM-NKX3.1 expression vectors, respectively. **C.** LNCaP cells were transfected with the HM control vector or the HM-NKX3.1 for 24 h and the cells were split into E-plates to analyze surface coverage before and after the CM treatments (CM including 250 or 500 pg/ml TNFα for 24 h.). Xcelligence real-time platform was used. The time of 0 indicates when the application of the CM was performed. **D.** The upregulation of β-catenin is associated with an increase in expression of c-myc and cyclin D1 in chronic CM treatments (62 or 125 pg/ml TNFα for 4 weeks). Consistently, this observation correlates with an increase in p-Akt^(S473)^ level and a decrease in p-β-catenin^(S33)^ in 4 wks of CM treatment. Black arrows indicate that the cellular boundaries of enlarged cells in comparison to control cells (white arrows). Two independent experiments were performed, and the blots were repeated at least three times.

NKX3.1 is a component of transcriptional repressor complex Groucho, which represses TCF4/β-catenin transcriptional activity [23], and is known as an oxidative damage regulator in prostate [17], [18]. Therefore, we examined putative role of NKX3.1 in β-catenin signaling after CM treatment (including 500 pg/ml TNFα, for 6 or 24 h). We found that ectopic NKX3.1 expression reduced the overall stability of β-catenin by suppressing Akt^(S473)^ phosphorylation and consistently restored GSK3β^(S9)^ and β-catenin^(S33)^ but not β-catenin^(S552)^ phosphorylation. Subsequently, the β-catenin transcriptional targets c-myc, cyclin D1 and MMP2 were downregulated both in the control and in the 6 h CM-treated samples (Figure 3A). Furthermore, immunoprecipitation of β-catenin followed by immunoblotting for E-cadherin showed that the β-catenin-E-cadherin association was partially restored both in the control and 6 h CM-treated cells expressing NKX3.1 (Figure 3B). To determine the regulatory role of NKX3.1, cell growth was also examined using the real-time assay in LNCaP cells after treatment with CM including two doses of TNFα (250 or 500 pg/ml). In cells expressing NKX3.1, the CM-mediated morphological changes were reversed remarkably and the cell growth was suppressed even under CM treatments (Figure 3C). These results imply that NKX3.1 has important functions in regulating cell morphology – perhaps taking a suppressive role on the epithelial-mesenchymal transition – and growth that are abrogated under inflammatory like conditions. Thus, NKX3.1 has an important function in controlling cell growth by regulating the β-catenin signaling and partially maintains plasma membrane localization of β-catenin at normal boundaries thereby stabilizing the β-catenin-E-cadherin association.

### Chronic CM Exposure Stabilizes β-Catenin Level

To study the effects of chronic exposure of inflammatory microenvironment, LNCaP cells were fed with lower doses (i.e. 62 or 125 pg/ml) of CM including TNFα for longer periods of time (4 weeks). Consistent with our findings in the acute model, Akt^(S473)^ phosphorylation increased following low dose but prolonged exposure to CM. As the p-β-catenin^(S33)^ was decreased and the total β-catenin accumulated, target genes c-myc and cyclin D1 were significantly upregulated (Figure 3D). Thus, these alterations facilitated the subsequent changes of prostate cells.

### CM-Mediated Migration of LNCaP Cells Is Suppressed by NKX3.1 Expression

**Figure 4:**
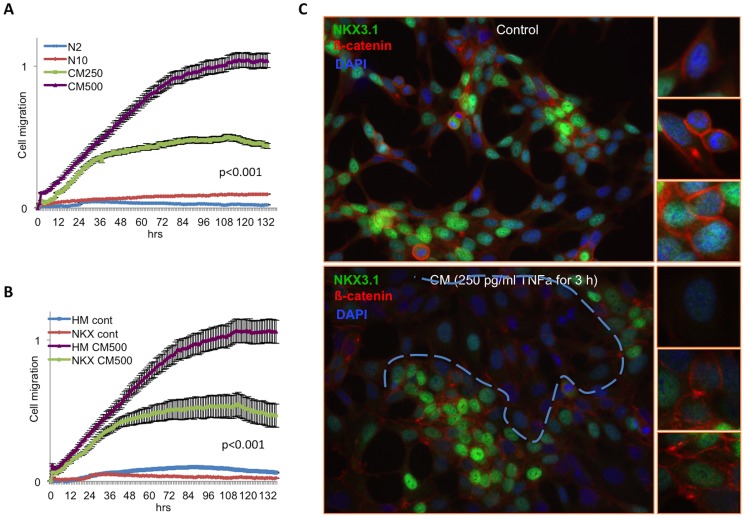
The CM treatment increases LNCaP cell migration in the Xcelligence CIM-plate. Additionally, induced migration of the LNCaP cells is positively correlated with the dose of inflammation that was examined using the real-time migration assay. **A.** N2: Negative control; medium containing 2% FBS was placed in both the upper and lower chambers. N10: Chemo-attractant control; medium containing 2% FBS was placed in the upper chamber, and medium containing 10% FBS was placed in the lower chamber of the CIM-plate. **B.** Ectopic NKX3.1 expression suppresses the inflammatory microenvironment-mediated migration of the LNCaP cells (green line). HM-Vec and HM-NKX3.1 indicate the control and the HisMax-NKX3.1 expression vectors, respectively. **C.** The cells exhibit clear membrane-localized β-catenin (upper panel). Although, membrane localized β-catenin level remains higher in cells that are expressing NKX3.1 at high levels, the cells responded to CM (250 pg/ml for 3 h) treatments and promoted the variable expression/localization of β-catenin correlated with remarkably variable NKX3.1 expression (lower panel). Blue dashed line indicates the region with depleted NKX3.1 expression, where β-catenin is also decreased especially at membrane boundaries.

To study the migration of LNCaP cells real-time Boyden chamber based migration assay was used (Xcelligence system). In this assay, FBS was used as a chemo-attractant, and 10% normal serum (N10) was used for positive control. We then used 2% FBS (N2) in the upper chamber of the CIM plate. As a negative control, 2% FBS (N2) was placed into both chambers. When the LNCaP cells were treated with the CM (including 250 or 500 pg/ml TNFα for 3 h and split onto the CIM-plate) the migration ability of the cells were correlated with increasing CM doses significantly (p<0.001) (Figure 4A). Additionally, the role of NKX3.1 expression in cell migration was examined and found that NKX3.1 significantly (p<0.001) suppressed the CM-induced migration of the LNCaP cells (Figure 4B). In order to understand how β-catenin stabilization correlates with NKX3.1 expression level, we also treated cells with 250 pg/ml TNFα containing CM that is enough for NKX3.1 degradation, and co-stained with β-catenin and NKX3.1. The data revealed that β-catenin stabilizes at membrane localizations with ectopic NKX3.1 expression and is disrupted with CM, concurrent to loss of NKX3.1 expression (Figure 4C). Thus, the data suggested that the NKX3.1 is an important factor for β-catenin localizations upon inflammation.

### Prostatic Inflammatory Atrophy (PIA) Inherits Prostatic Intraepithelial Neoplasia (PIN) from the Same Gland

**Figure 5:**
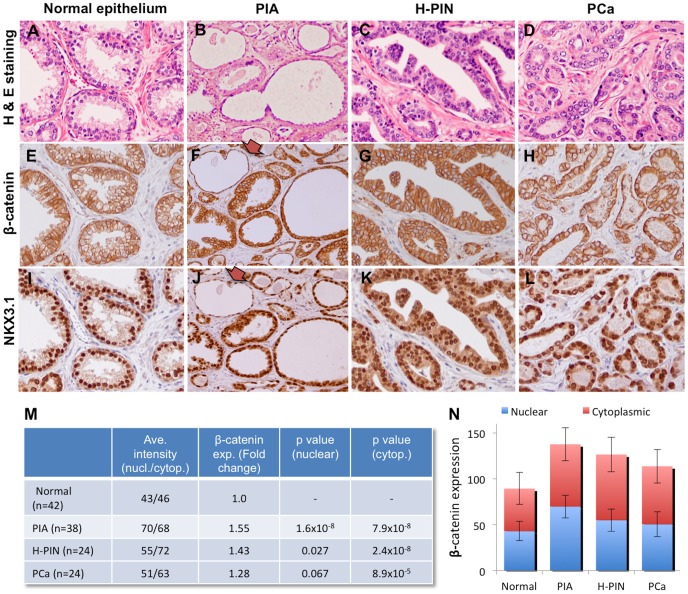
The CM treatment increases cytoplasmic β-catenin accumulation correlating with loss of nuclear NKX3.1 expression. Tissue sections (containing normal, PIA, H-PIN and PCa regions n = 42, 38, 24 and 24 respectively) were cut from 14 radical prostatectomy specimens and analyzed. The adjacent sections were stained with hematoxylin-eosin dye (**A–D**), β-catenin (**E–H**) and NKX3.1 (**I–L**) antibody to correlate the expression changes in *in vivo* samples. While glands from the normal prostate exhibited nuclear staining for β-catenin similar to PCa, the atrophy, H-PIN and PCa regions demonstrated remarkable increases in cytoplasmic staining. The representative images were taken from normal glands (**A, E, I**) the atropy (**B, F, J**), H-PIN lesions (**C, G, K**) and prostate adenocarcinoma (**D, H, L**) samples. The relative intensity from analyzed sections and statistical significance values were also given in comparison to normal sections (**M**). Histogram plot shows the variation of β-catenin expression between stages (**N**). The images were taken with a 20× objective. Also, brown colored arrows show the atrophy glands.

**Figure 6:**
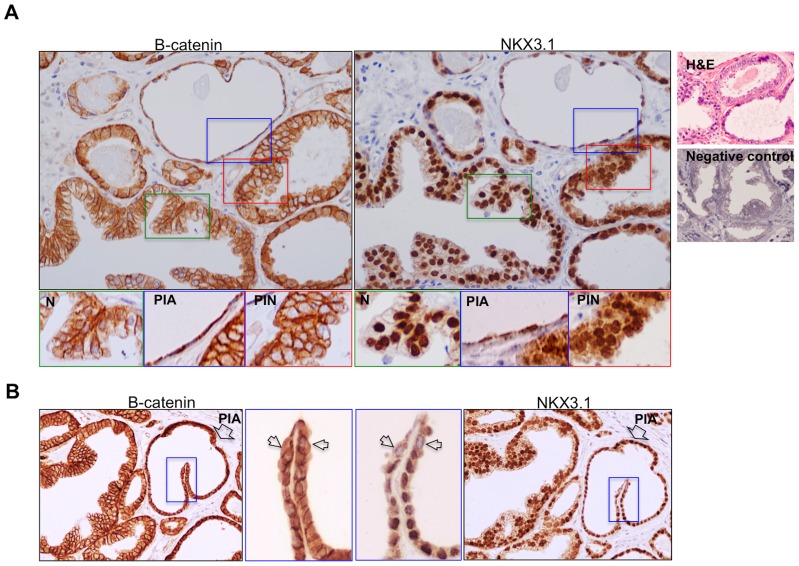
NKX3.1 and β-catenin expression variations might be related to macrophage infiltration in tissues consequent to inflammation. Tissues adjacent to the sections were used for β-catenin IHCs, and also used for NKX3.1 particularly in PIA and PIN regions. **A.** The sections were stained with hematoxylin-eosin dye and an NKX3.1 antibody. While these samples display stromal macrophage infiltration in most of the tissues adjacent to the normal and PIA regions, and not significant in PIN, broadly distributed NKX3.1 expression can be seen in PIN regions. High but not solely nuclear NKX3.1 expression was also shown in PIA and PIN in comparison to normal regions. **B.** Block arrows indicate the PIA gland and the small arrows show the cells with loss of NKX3.1 expression. The images were taken using 20× objective and also digitally magnified (smaller panels with blue rectangles). Negative (no Ab) staining controls were also provided.

Human radical prostatectomy specimens from 14 patients with prostatic inflammatory disease and prostate cancer were examined. The sample sizes were (42, 38, 24 and 24) for normal, proliferative inflammatory atrophy (PIA), H-PIN and cancer samples respectively. We examined these tissue sections for β-catenin and NKX3.1 expression using immunohistochemistry (IHC) staining. β-catenin was found uniformly expressed in the normal prostate epithelium with a remarkably low cytoplasmic expression with apparent membrane localization in the histological sections. However, the sections from different stages of prostate pathology exhibited a substantial increase (average expression values for normal: 46, PIA: 68, PIN: 72 and cancer: 63) in cytoplasmic β-catenin level when compared to normal epithelium (Figure 5). As, these significant increases (t-test values are given in Figure 5M) in β-catenin expression strongly associate with PIA, H-PIN and adenocarcinomas, cytoplasmic β-catenin levels are found remarkably high in atrophic glands (Figure 5N). Furthermore, the luminal cells in normal glands exhibited highly nuclear localization of NKX3.1, which was altered to cytoplasmic accumulation in cells from regions exhibiting PIA, PIN and PCa morphology. Also, partial or complete loss of NKX3.1 expression was demonstrated in some of the PIA regions in comparison to normal prostate epithelium (Figure 6) as it was previously reported [24]. Therefore, we suggest that cytoplasmic β-catenin accumulation subsequent to loss of functional NKX3.1 (Figure 6A) correlates with prostatic inflammatory atrophy, most likely facilitates the tumor heterogeneity (Figure 6B) and initiation that can be suppressed by androgen regulated NKX3.1 expression in prostate.

## Discussion

β-catenin, one of the major components of cell-cell adhesion plays a crucial role in many aspects of cell function and development [25], [26]. β-catenin localizes to different cellular compartments, including the plasma membrane, cytoplasm and nucleus, to form distinct complexes with various function. In addition to its activation by Wnt or activating mutations in Wnt signaling pathway, Wnt-independent signaling is also involved in the regulation of β-catenin transactivation [27], [28]. For example, β-catenin-TCF/LEF-1 signaling can be activated by growth factors EGF, HGF, insulin-like growth factor (IGF)-I, IGF-II, and insulin [29], [30]. However, EGF-induced β-catenin nuclear accumulation does not facilitate a detectable change in its phosphorylation by GSK3β or the half-life of β-catenin [9], [31]. This implies that GSK3β-independent or suppressed regulation might play a prominent role in EGF-induced β-catenin transactivation. Therefore, switching the function of β-catenin between cell-cell adhesion and Wnt pathway might be crucial for maintaining normal cellular function.

**Figure 7:**
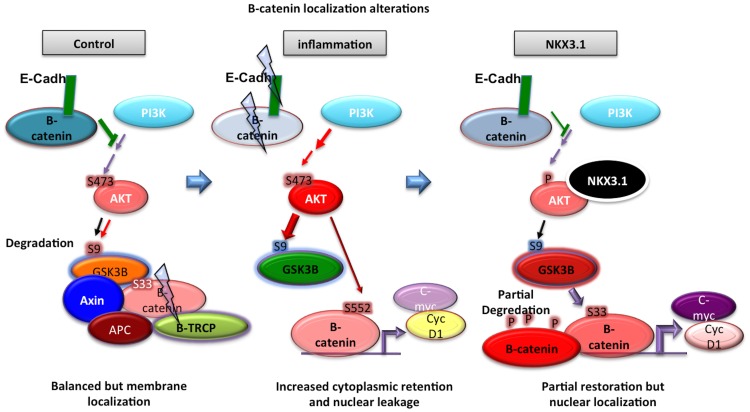
The CM exposure influences prostate cell progression through increasing cytoplasmic accumulation of β-catenin, which subsequently leads increased migration. Further, inflammation-mediated Akt activity and subsequent β-catenin transactivation can be deregulated by androgen responsive factor NKX3.1, stabilizing the p-β-catenin^(S33)^ pool, eventually influencing the maintenance of the intact β-catenin/E-cadherin association at the plasma membrane.

The deregulation of β-catenin function may promote tumorigenesis by altering gene transcription, increasing cell migration and abrogating cell polarity [11]. Thus, β-catenin contributes to prostate carcinogenesis at least in two ways. First, enhanced nuclear translocation of β-catenin results in increased proliferation, as seen in other cancer types. Second, tissue-specific molecular changes may dominate during tumorigenesis. Likewise, activation of the androgen receptor (AR) transactivation function is promoted by nuclear localization of β-catenin in prostate cells. Therefore, tissue-specific transcription factors (TCFs) and AR crosstalk with β-catenin may contribute to the progression of prostate hyperplasia, cell differentiation and tumorigenesis in prostate [2], [32]. Additionally, β-catenin stabilization and nuclear localization result in the upregulation of the β-catenin target genes cyclin D1 and c-myc, which can lead to the formation of the prostatic intraepithelial neoplasia (PIN)-like phenotype [33]. Further, activated Akt stabilizes β-catenin via inhibition of GSK3β [34] and directly phosphorylates β-catenin at S552, thereby promotes β-catenin transactivation [9]. Based on previous reports [35] and our findings reported here, we suggest that mimicking TNFα-mediated inflammation [20] in prostate cells results in a significant increase in pAkt^(S473)^, which consequently inhibits GSK3β kinase activity by increasing the phosphorylation from (S9) residue (Figure 1E). Furthermore, the decrease in β-catenin^(S33)^ phosphorylation implies that either proteosomal degradation is activated and β-catenin^(S33)^ is depleted by ubiquitination dependent proteosomal machinery, or there can be an increase in the stabilizing phosphorylation of β-catenin^(S552)^, which suppresses the S33 phosphorylation (Figure 1E) by enhanced Akt activity. As the majority of the total β-catenin localizes at the cell membrane, and associates with E-cadherin at adherent junctions, whereas the total β-catenin level does not change, we suggest that CM-mediated Akt activation abrogates the E-cadherin and β-catenin association at plasma membrane localization. This hypothesis is confirmed by the increasing ratios of nuclear/cytoplasmic and cytoplasmic/membrane localization of β-catenin in our studies and the schema was drawn accordingly (Figure 7). Moreover, in a previous study, Lamb et. al. [36] have demonstrated that blocking E-cadherin leads to a decrease in AKT activation. This data suggests that cell-cell adhesion is mediated by E-cadherin interaction that promotes the secretory-like cell survival through PI3K signaling. Hence, the putative mechanism can be a crosstalk between Akt signaling and E-cadherin localization, and its expression does not change in CM, but with ectopic NKX3.1 expression, E-cadherin localization to cell membrane might be facilitated through EGFR pathway. Ascertain the putative mechanism requires further studies.

As adherent junction components, E-cadherin and β-catenin have been extensively studied in models of tumor invasion and development, these molecules are phosphorylated by many kinases such as CK2, Src, Abl, Fer, and Fyn, which subsequently affect the adherent association of the cell membrane [37]–[39]. To investigate how CM-mediated Akt activity is crucial in regulating β-catenin^(S552)^ phosphorylation and contributes to the disassembly of β-catenin from membrane, we studied the ectopic expression of androgen-regulated NKX3.1 with CM treatments. As the NKX3.1 is an intracellular Akt kinase regulator in prostate cell [40], it was previously reported that it might prevent prostate cancer initiation by stabilizing p53 and inhibiting Akt [41]. Consistently, loss of NKX3.1 results deregulated Akt function, which directly phosphorylates β-catenin in prostate cells. Despite, we found that NKX3.1 reversed the CM-mediated migration of LNCaP cells, facilitated in a substantial improvement in β-catenin degradation and clearly suppressed the proliferation and invasive effects of β-catenin in prostate cells. This might be related to its tumor suppressor function by suppressing the activating phosphorylations of Akt concurrent to facilitating the transactivation of oxidative stress scavengers in prostate. Further, the data from the real-time assays (Figure 1B and 3C) demonstrate that the morphological changes occur 3 h after inflammation in these cells. Since, this is a quite short time frame for β-catenin stabilization and subsequent proliferation, we propose that the dissociation of β-catenin from membrane disrupts E-cadherin association at adherent junctions, might occur immediately upon cytokine exposure thereby promotes migration. These results demonstrate that the increased migration correlates with expression of c-myc and cyclin D1, which advance uncontrolled proliferation during late-stages of carcinogenesis.

Moreover, the observations obtained from cell line studies suggested that the β-catenin localization increased substantially in cytoplasm after 6 h of CM treatment and this was confirmed in human prostate tissues from patients with prostatic inflammatory disease. Further, the *in vivo* study has indicated that some of the atrophic glands, which inherit PIN lesions, are in close proximity to adenocarcinomas, exhibit extensive E-cad expression and increased Akt^(S308)^ priming phosphorylations (data not shown). Consistent with the previous report [42] these glands also exhibited a substantial loss of membrane-bound β-catenin and partial loss of NKX3.1 expression. Consequently, the loss of β-catenin expression at the cytoplasmic borders implies that the reduced levels of β-catenin evade interaction with E-cadherin in these PIA regions. Thus, prostatic inflammation may facilitate the progression of prostate cancer in PIA glands and this process presumably involves the loss of protective functions of important mediators of cells, such as functional NKX3.1 as well as B-catenin in plasma membrane.

As the effects of β-catenin on tumorigenesis, invasion and metastasis of prostate cells have been reported previously, increased translocation of β-catenin upon Wnt signaling has also been detected in 25–38% of castration-resistant metastatic prostate cancers [38]. Furthermore, transcriptionally active β-catenin may interact with TCF to induce tumorigenic proliferation with or without AR, indicating that this mechanism might be independent of androgens [32], [43]. Although β-catenin is regulating the self-renewal capacity of prostate cells independent of AR, the ligand-dependent suppressive function of AR enhances the regulation of β-catenin function [32]. Therefore, we suggest that activated β-catenin signaling may play an important role in prostate tumorigenesis, particularly when AR function is lost concurrent to increased Akt phosphorylations (data not shown) due to inflammatory cytokine exposure [20].

Furthermore, the interaction between the adhesion and transcriptional activity of β-catenin has been intensely investigated in a recent study [44]. In this study, Maher et. al., have suggested that the cell-cell adhesion is controlled by cadherins, and the epithelial–mesenchymal transition (EMT) is characterized by the loss of cell–cell adhesion besides increased cell motility, which are well-known alterations that occur during the development of carcinomas. Consistent with previous reports and our findings, E-cadherin function is lost and the cadherin-catenin complex is dissociated from the membrane with inflammation (similar to EMT). Consequently, increased b-catenin transactivation correlates with the loss of E-cadherin-mediated cell adhesion [11]. Taken together, we suggest that cytokine exposure with CM treatment (inflammatory like microenvironment) promotes the migratory ability of cells by influencing both functions of β-catenin i.e., enhancing the transactivation function and abrogating the association with E-cadherin. This regulation of β-catenin can be partially restored through the protective role of NKX3.1; the mechanism needs to be investigated in detail along with other mechanisms that contribute to EMT like phenotype.

## Supporting Information

Figure S1 **This figure demonstrates the increased E-cad and p-Akt(S308) levels in human samples especially in PIA (red rectangles) and PIN (blue rectangles) regions.** The block arrows indicate that the cells with increased p-Akt(S308) level in PIA regions, suggesting that the growth and the expression heterogeneity is higher in PIA glands than normal glands.
(TIF)
