# Supplementary figures and images for: TNFα-Mediated Loss of β-Catenin/E-Cadherin Association and Subsequent Increase in Cell Migration Is Partially Restored by NKX3.1 Expression in Prostate Cells
Source: PLoS One. 2014 Oct 31;9(10):e109868. doi: 10.1371/journal.pone.0109868 (PMC4215977; doi:10.1371/journal.pone.0109868)

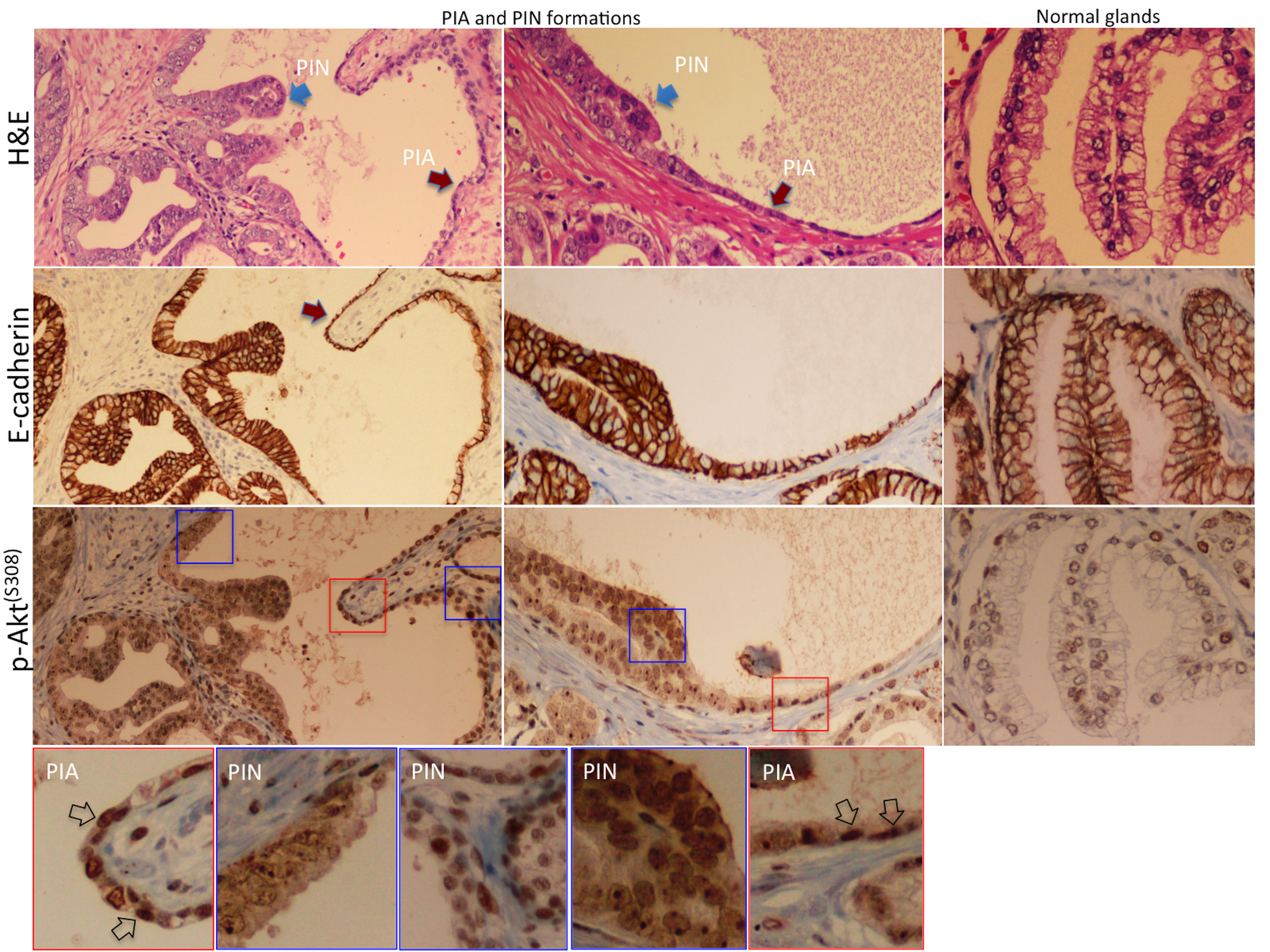

Supplement: Figure S1 — This figure demonstrates the increased E-cad and p-Akt(S308) levels in human samples especially in PIA (red rectangles) and PIN (blue rectangles) regions. The block arrows indicate that the cells with increased p-Akt(S308) level in PIA regions, suggesting that the growth and the expression heterogeneity is higher in PIA glands than normal glands. (TIF) [file pone.0109868.s001.tif]
